# Supplementary material for: End Sequence Analysis Toolkit (ESAT) expands the extractable information from single-cell RNA-seq data
Source: Genome Res. 2016 Oct;26(10):1397–410. doi: 10.1101/gr.207902.116 (PMC5052061; doi:10.1101/gr.207902.116)
Supplement: Supplemental Material [file supp_gr.207902.116_Supp_Materials.tar.gz › ESAT-esat_v0.1/doc/html/functions_vars_o.html]

ESAT: Class Members - Variables


ESAT


- Main Page
- Related Pages
- Packages
- Classes
- Files

- Class List
- Class Index
- Class Hierarchy
- Class Members

- All
- Functions
- Variables
- Enumerator

- a
- b
- c
- d
- e
- f
- g
- h
- i
- k
- l
- m
- n
- o
- p
- q
- r
- s
- t
- u
- v
- w


All Classes Namespaces Files Functions Variables Enumerator Pages

### - o -

- offset
  : broad.core.sequence.AbstractFastaParser
- ORIGINAL\_ARRAY\_LEFT
  : broad.pda.seq.rap.CountProbes
- ORIGINAL\_ARRAY\_RIGHT
  : broad.pda.seq.rap.CountProbes
- OUTER\_LENGTH
  : broad.pda.seq.rap.PlotAggregateRegions
- OUTPUT
  : broad.pda.samtools.FilterPairedReadsByMAQ
  , broad.pda.samtools.PairedEndTDF
  , broad.pda.samtools.TrimFastqReads
  , broad.pda.seq.rap.BuildRatioNullDistribution
  , broad.pda.seq.rap.CalculateCoverageStats
  , broad.pda.seq.rap.CollectAnnotationEnrichments
  , broad.pda.seq.rap.CountProbes
  , broad.pda.seq.rap.CountReads
  , broad.pda.seq.rap.DistanceToBED
  , broad.pda.seq.rap.GCContent
  , broad.pda.seq.rap.PlotAggregateRegions
  , broad.pda.seq.rap.RatioPermutationPeakCaller
  , broad.pda.seq.rap.rna.CountReads
  , broad.pda.seq.rap.ShuffleBAM
  , broad.pda.seq.rap.ShuffleBED
  , broad.pda.seq.rap.SkellamKLPeakCaller
  , broad.pda.seq.rap.SlideAndCalculate
  , broad.pda.seq.rap.SlideAndCount
  , broad.pda.seq.rap.UnmarkDuplicates
- OUTPUT\_1
  : broad.pda.samtools.FilterFastqReads
- OUTPUT\_2
  : broad.pda.samtools.FilterFastqReads
- OVERLAP
  : broad.pda.seq.rap.BuildRatioNullDistribution
  , broad.pda.seq.rap.CountProbes
  , broad.pda.seq.rap.PlotAggregateRegions
  , broad.pda.seq.rap.RatioPermutationPeakCaller
  , broad.pda.seq.rap.SkellamKLPeakCaller
  , broad.pda.seq.rap.SlideAndCalculate
  , broad.pda.seq.rap.SlideAndCount


---

Generated on Fri Aug 15 2014 15:11:17 for ESAT by  

 1.8.7
